# Crosstalk between cancer-associated fibroblasts and immune cells in peritoneal metastasis: inhibition in the migration of M2 macrophages and mast cells by Tranilast

**DOI:** 10.1007/s10120-021-01275-5

**Published:** 2022-01-07

**Authors:** Yusuke Nakamura, Jun Kinoshita, Takahisa Yamaguchi, Tatsuya Aoki, Hiroto Saito, Toshihide Hamabe-Horiike, Shinichi Harada, Sachiyo Nomura, Noriyuki Inaki, Sachio Fushida

**Affiliations:** 1 Department of Gastroenterological Surgery, Graduate School of Medical Science, Kanazawa University, 13-1 Takara-machi, Kanazawa, Ishikawa 920-8641 Japan; 2 Center for Biomedical Research and Education, School of Medicine, Kanazawa University, Kanazawa, Japan; 3 Department of Gastrointestinal Surgery, Graduate School of Medicine, The University of Tokyo, Tokyo, Japan

**Keywords:** Peritoneal metastasis, Cancer-associated fibroblast, M2 macrophage, Mast cell, Tranilast

## Abstract

**Background:**

The role of tumor–stroma interactions in tumor immune microenvironment (TME) is attracting attention. We have previously reported that cancer-associated fibroblasts (CAFs) contribute to the progression of peritoneal metastasis (PM) in gastric cancer (GC), and M2 macrophages and mast cells also contribute to TME of PM. To elucidate the role of CAFs in TME, we established an immunocompetent mouse PM model with fibrosis, which reflects clinical features of TME. However, the involvement of CAFs in the immunosuppressive microenvironment remains unclear. In this study, we investigated the efficacy of Tranilast at modifying this immune tolerance by suppressing CAFs.

**Methods:**

The interaction between mouse myofibroblast cell line LmcMF and mouse GC cell line YTN16 on M2 macrophage migration was investigated, and the inhibitory effect of Tranilast was examined in vitro. Using C57BL/6J mouse PM model established using YTN16 with co-inoculation of LmcMF, TME of resected PM treated with or without Tranilast was analyzed by immunohistochemistry.

**Results:**

The addition of YTN16 cell-conditioned medium to LmcMF cells enhanced CXCL12 expression and stimulated M2 macrophage migration, whereas Tranilast inhibited the migration ability of M2 macrophages by suppressing CXCL12 secretion from LmcMF. In PM model, Tranilast inhibited tumor growth and fibrosis, M2 macrophage, and mast cell infiltration and significantly promoted CD8 + lymphocyte infiltration into the tumor, leading to apoptosis of cancer cells by an immune response.

**Conclusion:**

Tranilast improved the immunosuppressive microenvironment by inhibiting CAF function in a mouse PM model. Tranilast is thus a promising candidate for the treatment of PM.

## Introduction

Gastric cancer is one of the most common malignancies in East Asia, and peritoneal metastasis (PM) has a poor prognosis with a 1-year survival rate of approximately 40% [1, 2]. This poor prognosis might be due to the poor delivery of systemic chemotherapy, attributed to the blood-peritoneal barrier and high intra-tumor pressure [3, 4]. Thus, repeated intraperitoneal chemotherapy using taxanes (paclitaxel or docetaxel) with systemic chemotherapy has been performed in the last decade with a 1-year survival rate of 70–80% [5–8]. However, it is difficult to cure patients with PM. We previously clarified that the tumor immune microenvironment (TME) in PM differs from that in primary lesions [9], exhibiting an immune suppressive state with low infiltration of CD8^+^ cells and high infiltration of M2 macrophages by various cytokines or chemokines from cancer-associated fibroblasts (CAFs) [10]. These immunosuppressive states lead to resistance not only to immune checkpoint inhibitors (ICIs) but also to chemotherapy. Additionally, PM characterized by abundant fibrous stroma is considered to be caused by crosstalk between cancer cells and CAFs with Transforming growth factor β (TGF-β), or between mast cells and CAFs by interleukin-17A (IL-17A) [11]. This fibrosis induces occlusion of the luminal organs, such as intestinal obstruction, hydronephrosis, and obstructive jaundice, and interferes with the delivery of chemotherapeutic agents due to its high intra-tumor pressure [4]. Therefore, it is necessary to suppress the function of CAFs and improve the TME and tumor fibrosis.

As a preclinical study, we established a peritoneal metastatic model co-inoculated with mouse gastric cancer cells YTN-16 and mouse myofibroblasts LmcMF using immunocompetent mice [9]. This peritoneal tumor showed invasive progression, rich fibrosis, low infiltration of CD8^+^ cells, and high infiltration of M2 macrophages, which is similar to the clinical features of PM. Recently, synaptotagmin XIII (SYT13) was considered a candidate molecule for PM formation due to its expression at significantly higher levels in patients with PM but not in those with hepatic or lymphatic metastasis [12]. This malignant effect of SYT13 signaling, represented by the migration and invasion of cancer cells, is mediated by its interaction with C–X–C motif chemokine ligand 12 (CXCL12) [13]. Kanda et al. demonstrated that amido-bridged nucleic acid-modified antisense oligonucleotides targeting SYT13 inhibit migration and invasion of GC cells, resulting in decreased peritoneal nodules in a mouse model [14]. Although these antisense oligonucleotide therapies are promising, a clinical application might be difficult in fibrous PM, with poor delivery due to their large molecular size.

Tranilast, an anti-allergy drug, inhibits fibroblast growth and the production of chemical mediators from fibroblasts or mast cells [14, 15]. Accordingly, Tranilast may also suppress the secretion of chemokines, such as CCL2 or CXCL12, from CAFs and cytokines, such as IL-17A from mast cells [10, 11]. There are several reports of Tranilast as an anticancer drug by suppressing the synthesis of TGF-beta from cancer cells and fibroblasts [16, 17]. We also previously reported that Tranilast suppresses TGF-β/Smad signaling by inhibiting the phosphorylation of Smad2/3, resulting in decreased tumor growth and fibrosis of subcutaneous tumors in nude mice [18]. Therefore, Tranilast may improve tumor fibrosis and the immune suppressive microenvironment by blocking the migration of mast cells and macrophages, even in the PM of GC patients.

In this study, we demonstrated that Tranilast interfered with the migration of M2 macrophages and mast cells, decreased fibrosis, and increased CD8^+^ cell infiltration in peritoneal tumor co-inoculated YTN-16 cells and LmcMF cells using a C57BL/6 J immunocompetent mouse model.

## Materials and methods

### Cell lines and cell culture

Bone marrow-derived macrophages were differentiated from bone marrow cells under incubation with macrophage-colony stimulating factor (40 ng/mL) for 7 days, followed by incubation with IL-4 (20 ng/mL) for 2 days in RPMI medium containing 10% fetal bovine serum (FBS), penicillin (100 U/mL), and amphotericin B (2 μg/mL) at 37 °C in a 5% CO_2_ atmosphere [19]. The purity of double-positive cells for CD11b, a pan-macrophage marker, and CD163, an M2 macrophage marker, was confirmed using flow cytometry.

YTN16 is a transplantable gastric cancer cell line in C57BL/6 mice. YTN16 cells were established from subcutaneous tumors by injecting primary cultured cells derived from mouse gastric adenocarcinoma. Mouse gastric tumors were established in p53 heterozygous knockout C56BL/6 mice by adding N-methyl-N-nitrosourea to the animals’ drinking water [20]. The resulting tumor cells were cultured in high-glucose Dulbecco’s modified Eagle medium (DMEM, Sigma-Aldrich Japan, Tokyo, Japan) containing 1.0 mL/L MITO (Coning Japan, Tokyo, Japan), 10 mL/L L-glutamine, 10 mL/L penicillin/streptomycin, and 10% FBS on plastic dishes coated with Type I collagen solution (0.5% Atelocollagen Acidic Solution IPC-50; Koken Co., Ltd., Japan) at 37 °C in a 5% CO_2_ atmosphere.

The mouse intestinal myofibroblast cell line (LmcMF) derived from mouse colonic mucosa was established by Takashi Ohama and Koichi Sato, Laboratory of Veterinary Pharmacology, Joint Faculty of Veterinary Medicine, Yamaguchi University [21]. Cells were cultured in DMEM containing 10% FBS at 37 °C in a 5% CO_2_ atmosphere.

### Chemical

Tranilast (N-[3,4-dimethoxycinnamonyl]-anthranilic acid) was obtained from Tokyo Chemical Ind. Co. (Japan), diluted to the required concentrations in DMEM medium for the in vitro study, and dissolved in 1% NaHCO3 solution for the in vivo study.

### Migration assay of M2 macrophages

The effect of Tranilast on M2 macrophage migration ability was determined using a BD BioCoat Matrigel Invasion Chamber for 24-well plates (BD Bioscience, USA), according to the manufacturer’s instructions. First, matrigels were rehydrated using 750 μL of serum-free medium; LmcMF cells (1 × 10^5^ cells/well) were then seeded into the lower chamber with or without conditioned medium (CM) of YTN-16 cells. Next, 500 μL of M2 macrophages (5 × 10^4^ cells/well) in serum-free media with or without Tranilast (50 μM) were seeded into the upper chamber of the system. After 24 h of incubation, the cells in the upper chamber were removed, and the cells that had invaded through the matrigel membrane were stained with hematoxylin and fixed in 100% methanol. The membranes were removed from the inserts and mounted on slides. Invading cells were counted using a microscope at × 200 high-power magnification in several fields of triplicate membranes.

### Mouse multiplex cytokine/chemokine analysis

CM samples were collected from LmcMF alone, YTN-16 alone, direct and indirect co-culture with YTN16, and LmcMF; they were cultured in serum-free DMEM for 48 h, and the particulates were removed by filtration through a 0.2 μm filter. Each CM was analyzed to determine which chemokines were induced by interacting with YTN16 using a mouse multiplex cytokine/chemokine array (Proteome Profiler Mouse XL Cytokine Array, R&D systems, USA), allowing for 111 cytokines at a time, according to the manufacturer’s instructions. Chemiluminescent data were collected using the Gel Image system (ATTO, Light-Capture II) quantitated with CS analyzer software (Version 3.0) by measuring the intensities of the detected spots.

### Polymerase chain reaction analysis

Total RNA was extracted from LmcMF cells using the RNeasy Mini Kit (Qiagen, Hilden, Germany). RT-PCR was performed using a Multiplex Quantitative PCR system (Mx3000P; Agilent Technologies, Inc. CA, USA) using an SYBR Green PCR Kit (Qiagen) in triplicate using specific primers. The primer sequences used to determine the expression of the target genes were as follows:

- CCL2, forward: 5′-CTACCCCAACAATGCACCTT-3′ and reverse: 5′-GAGGACTTGCTGGACAGGAG-3′
- CXCL12, forward: 5′-TGCATCAGTGACGGTAAACCA-3′ and reverse: 5′-TGTCTGTTGTTCTTCAGCCGTGC-3′

### ELISA

CXCL12 levels in the culture media of LmcMF cells were quantified using a Mouse CXCL12 DuoSet ELISA kit (R&D Systems) according to the manufacturer’s instructions. LmcMF cells were cultured with or without CM of YTN-16 cells, and CXCL12 levels were measured 48 h after adding various Tranilast concentrations. The optical density of each well was read at 450 and 570 nm using a microplate reader (Multiskan GO, Thermo Scientific, USA). The CXCL12 concentration in each well was calculated from the absorbance values using a standard curve.

### Western blotting

CXCR4 expression was assessed by western blot analysis. Protein was blocked with blocking solution (0.1% Tween-20; EZ Block ATTO Corporation, Japan) and incubated with primary CXCR4 antibody (Invitrogen, 12G5, diluted 2 μg/ml), and anti-β-actin monoclonal antibody (AC-15, mouse monoclonal IgG, diluted 1:10,000; Sigma).

### Mouse allograft PM model and subcutaneous tumor model

The animal use proposal and experimental protocol (AP-183944) were reviewed and approved by the Animal Care and Use Committee of Kanazawa University. All animal experiments were performed in accordance with the standard guidelines of Kanazawa University. C57BL/6J, BALB/c, and BALB/c-nu/nu mice were purchased from Charles River Laboratories, Inc., Yokohama, Japan. For the mouse allograft PM model using C57BL/6J, YTN16 cells were co-cultured with an equivalent number of LmcMFs for 5 days, and a total of 1 × 10^7^ cells in 1 mL of high-glucose DMEM were then inoculated intraperitoneally. Two groups of six mice each were established: YTN16 cells co-inoculated with LmcMF cells, with or without Tranilast. In this study, total inoculated cell counts were aligned to compare tumor weights consisting of both cancer and stromal cells, including LmcMFs. Beginning on day 7, mice were administered 200 mg/kg/day of Tranilast daily by gavage. The animals were carefully monitored, and body weights were measured every 3 days. At day 21, the mice were killed, and tumors were removed for weight calculation and immunohistochemical examination. Peritoneal tumor collection was performed by a researcher who was blinded to treatment information. For the mouse subcutaneous tumor model using BALB/c and BALB/c-nu/nu, mice inoculated with 5 × 10^5^ cells of co-cultured YTN16 cells and LmcMF cells were treated with or without Tranilast from day 7 in the same manner as the PM model. Tumor growth was monitored, mice were killed on day 14, and excised tumors were examined by immunohistochemistry. Tumor growth was calculated using the formula (*L* × *W*^2^)/2, where *L* and *W* are the longest and shortest tumor diameters, respectively.

### Immunohistochemistry and immunofluorescence

Tumor specimens were fixed in 10% neutral-buffered formalin and embedded in paraffin. Sections were stained with hematoxylin and eosin (H&E), Azan stain for assessment of fibrosis, and toluidine blue stain for mast cells, while the expression of various antigens was assessed immunohistochemically. Deparaffinized sections were pretreated by autoclaving in 10% citric acid buffer at 120 °C for 15 min. After the treatment with protein block serum (Dako Co., Kyoto, Japan) for 10 min and incubation with 2% skim milk for 30 min to block nonspecific reactions, sections were incubated with primary antibody at 4 °C overnight. The following primary antibodies were used: αSMA (Abcam, ab5964, UK), CD163 (Abcam, ab182422), CD8 (Abcam, ab209775), and CD31 (BD Pharmingen, 553370, USA) antibodies. After the sections were washed in PBS, immunoreactivity was visualized using EnVision reagent (Dako Co.), and the slides were developed with diaminobenzidine and counterstained with hematoxylin.

All sections were examined using an Olympus UPLFLN 100X objective lens, DP70-SET microscope digital camera, and ‘Cell Scans Standard’ as the acquisition software. The degree of fibrosis was calculated as the percentage of fibrosis within the whole section of all samples using a BZ-9000 BZII microscope (Keyence, Japan). For immunofluorescence to detect apoptotic cells, anti-active caspase-3 (BD Pharmingen, 559565) was used as primary antibody. The secondary antibody used was anti-rabbit IgG antibody conjugated with Alexa Fluor^®^ 488 (Abcam, ab150073). Nuclei were counterstained using DAPI. The slides were observed using an immunofluorescence microscope (BX50/BS-FLA; Olympus, Japan).

### Quantification of immunostaining parameters

Data were obtained by manually counting positively stained cells in five non-overlapping intratumoral fields. Stained cells in mouse model tumors were accessed at × 100 magnification. All immunostaining was independently interpreted by two researchers (JK and TY).

### TdT-mediated dUTP nick end labeling (TUNEL) assay for apoptotic cell detection

The apoptotic cells of mice peritoneal tumors in each group were detected using the DeadEnd^™^ Fluorometric TUNEL System (Promega) according to the manufacturer’s instructions. Under a confocal microscope (Nikon, Tokyo, Japan), the nuclei were stained blue with DAPI, and the apoptosis-positive cells were stained green. Five high-power fields (× 100) were randomly selected from each group, and the number of apoptotic cells was counted. Apoptotic cell rate = the number of green cells/number of blue cells × 100 (%).

### Statistical analysis

Statistical analyses were conducted using SPSS statistical software, version 23 (IBM Corp., Armonk, NY, USA). Statistical analysis was performed using Student’s *t* test (two tailed). Statistical significance was set at *p* < 0.05. The results are presented as the mean ± SEM of three independent experiments.

## Results

### Transwell migration assay of macrophages

**Fig. 1:**
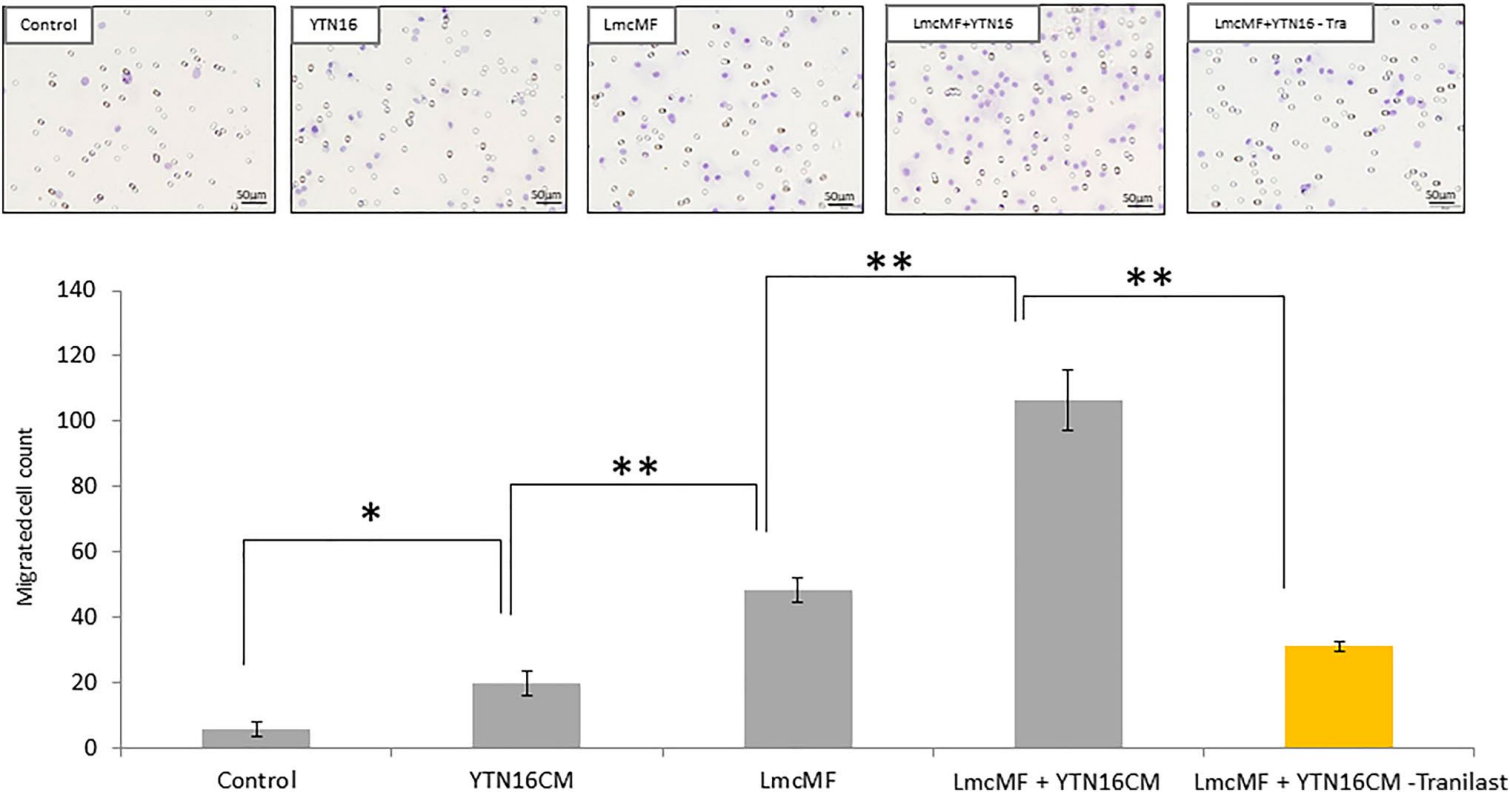
Co-culture of YTN16 cells with LmcMF cells promotes the migration of M2 macrophage. Transwell migration assay showed that the addition of YTN16 cell-conditioned medium (CM) to LmcMF cells enhanced M2 macrophage migration, while Tranilast administration (50 μM) significantly inhibited the migration of M2 macrophages stimulated by LmcMF cells and YTN16 cells-CM. Migrating cells were counted in four randomly chosen fields. Data represent the mean ± SEM of triplicate wells from three independent experiments (**p < 0.01)

We first evaluated the migration ability of M2 macrophages differentiated with macrophage-colony stimulating factor and IL-4 from mouse bone marrow cells using a transwell migration assay. The addition of YTN16 cells-CM to LmcMF cells significantly induced the migration ability of M2 macrophages compared to monoculture of YTN16 cells (106.5 ± 9.1 cells vs. 48.2 ± 3.9 cells, *p* < 0.01). 24 h after the addition of YTN16 cell-CM to LmcMF cells, the number of M2 macrophages penetrating to the lower surface of the transwell chamber was significantly decreased in the Tranilast-treated group compared to the control group (31.0 ± 1.4 cells vs. 106.5 ± 9.1 cells, *p* < 0.01) (Fig. 1).

### The interaction of LmcMF cells with YTN16 cells increases CXCL12 production levels

**Fig. 2:**
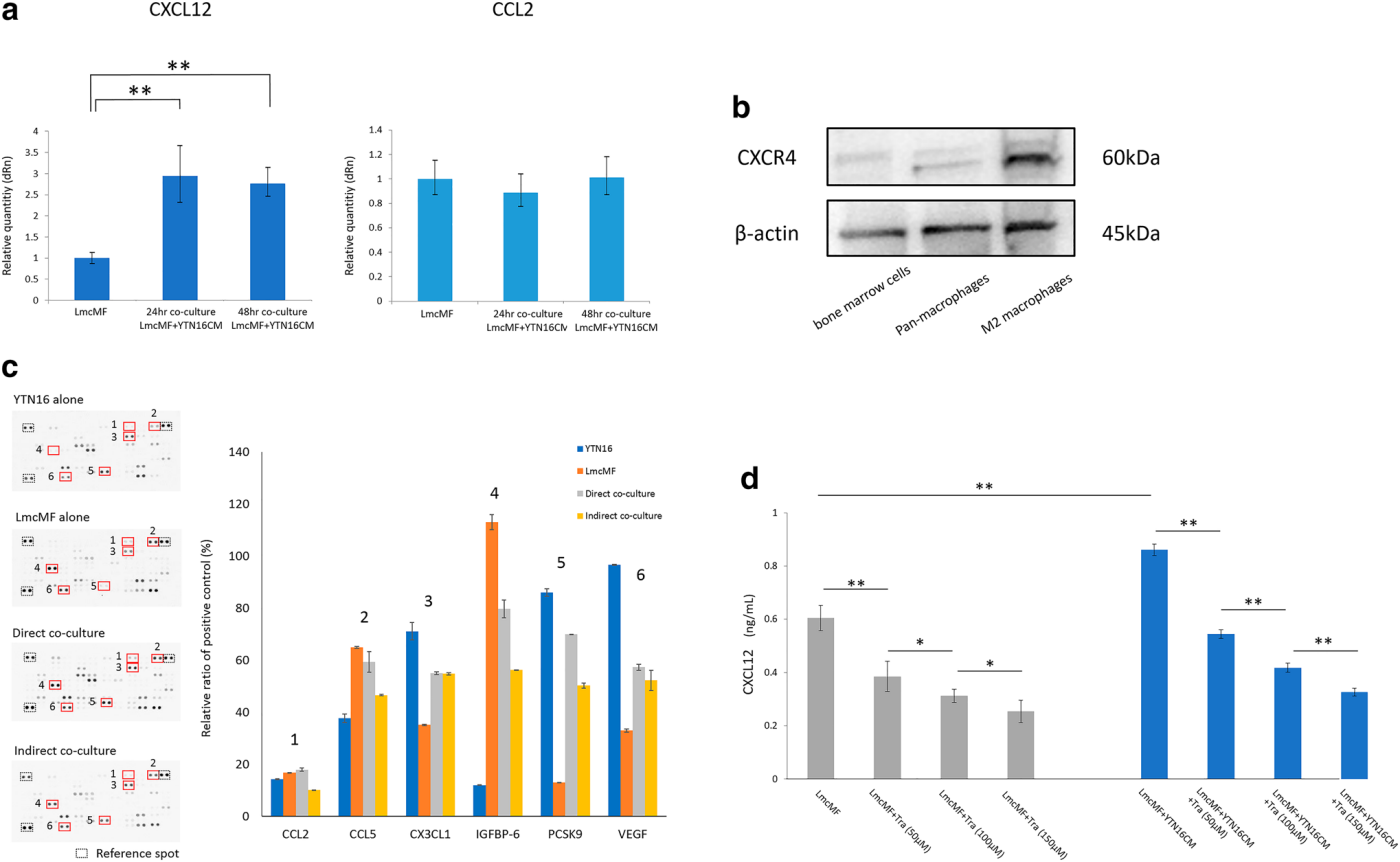
LmcMF cells enhance CXCL12 secretion when the co-culture with YTN16 cell-conditioned medium and Tranilast inhibits this effect. **a** LmcMF cells were cultured with conditioned medium (CM) of YTN16 cells for 48 h. CXCL12 and CCL2 mRNA expression levels in LmcMF cells cultured with YTN16 cells and CM were analyzed by PCR analysis. **b** The expression of CXCR4 in mouse bone marrow cells, pan-macrophages (after M-CSF stimulation), and M2 macrophages (after M-CSF and IL-4 stimulation). **c** Cytokine/chemokine arrays of YTN16 and LmcMF cells and direct/indirect co-culture supernatants: quantitative analysis of the relative levels of 111 factors. Values are normalized to positive control. The bar graphs show the representative chemokine values that changed in expression between samples, but no factors were upregulated by co-culture. **d** The concentrations of CXCL12 from LmcMF cells were examined by ELISA. Tranilast inhibited the secretion of CXCL12 from LmcMF cells and inhibited the upregulation of CXCL12 by the addition of YTN16 cells-CM in a concentration-dependent manner. Data represent the mean ± SEM of triplicate wells for three independent experiments (*p < 0.05, **p < 0.01). Tra: Tranilast

These results indicate that the effect of LmcMF on M2 macrophage migration potency may be mediated by paracrine effects. We then used RT-PCR to determine whether the expression of CCL2 and CXCL12, the major chemotactic factors of macrophages, from LmcMF cells was upregulated by adding the YTN16 cell-CM. As shown in Fig. 2a, LmcMF cells markedly upregulated the mRNA level of CXCL12 upon culture with YTN16 cells-CM. In contrast, the expression of CCL2 in LmcMF cells was not altered by YTN16 cells-CM. The expression of CXCR4 receptors for CXCL12 was confirmed in M2 macrophages by western blotting (Fig. 2b).

To define the other migration factors secreted from LmcMF cells to macrophages, the differences in cytokine expression between LmcMF cells, YTN16 cells, and LmcMF cells plus YTN16 cells were further examined using Multiplex cytokine/chemokine array. Of the 111 factors examined, no molecule including CCL2 was upregulated in the CM of co-culture with LmcMF and YTN 16 cells (Fig. 2c). These results suggest that CXCL12 from LmcMF cells is the most important chemotactic factor in macrophages. We then evaluated the expression of CXCL12 in the supernatant of LmcMF cells cultured alone and in the supernatant of LmcMF cells with addition of YTN16 cells-CM, with or without Tranilast. The results showed that Tranilast suppressed the expression of CXCL12 from LmcMF cells in a dose-dependent manner. In addition, the addition of YTN16 cells-CM promoted the expression of CXCL12 from LmcMF, and Tranilast inhibited this effect as well (Fig. 2d).

### Tranilast inhibited proliferation, angiogenesis and fibrosis in mouse PM tumor and mouse subcutaneous tumor

**Fig. 3:**
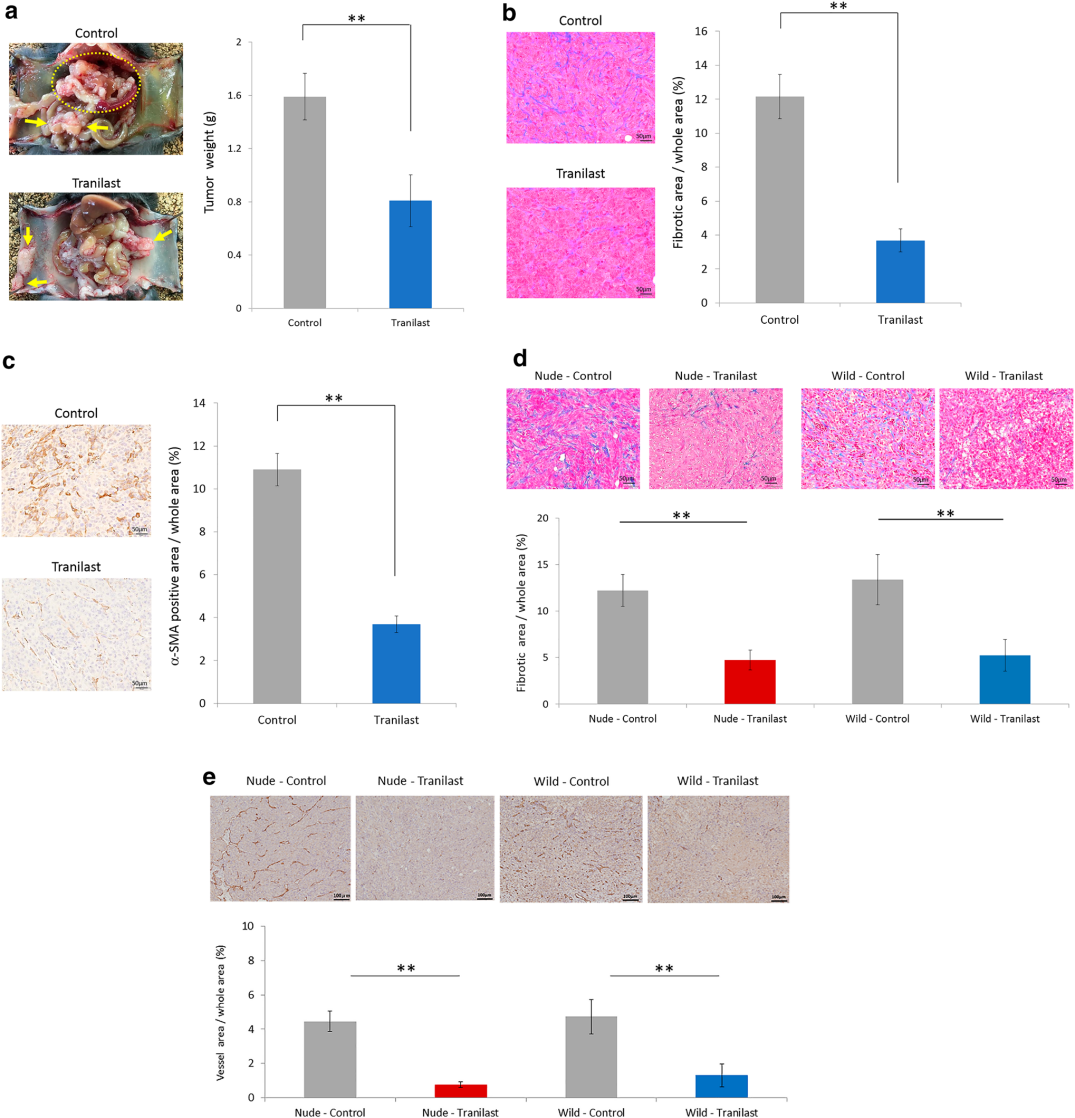

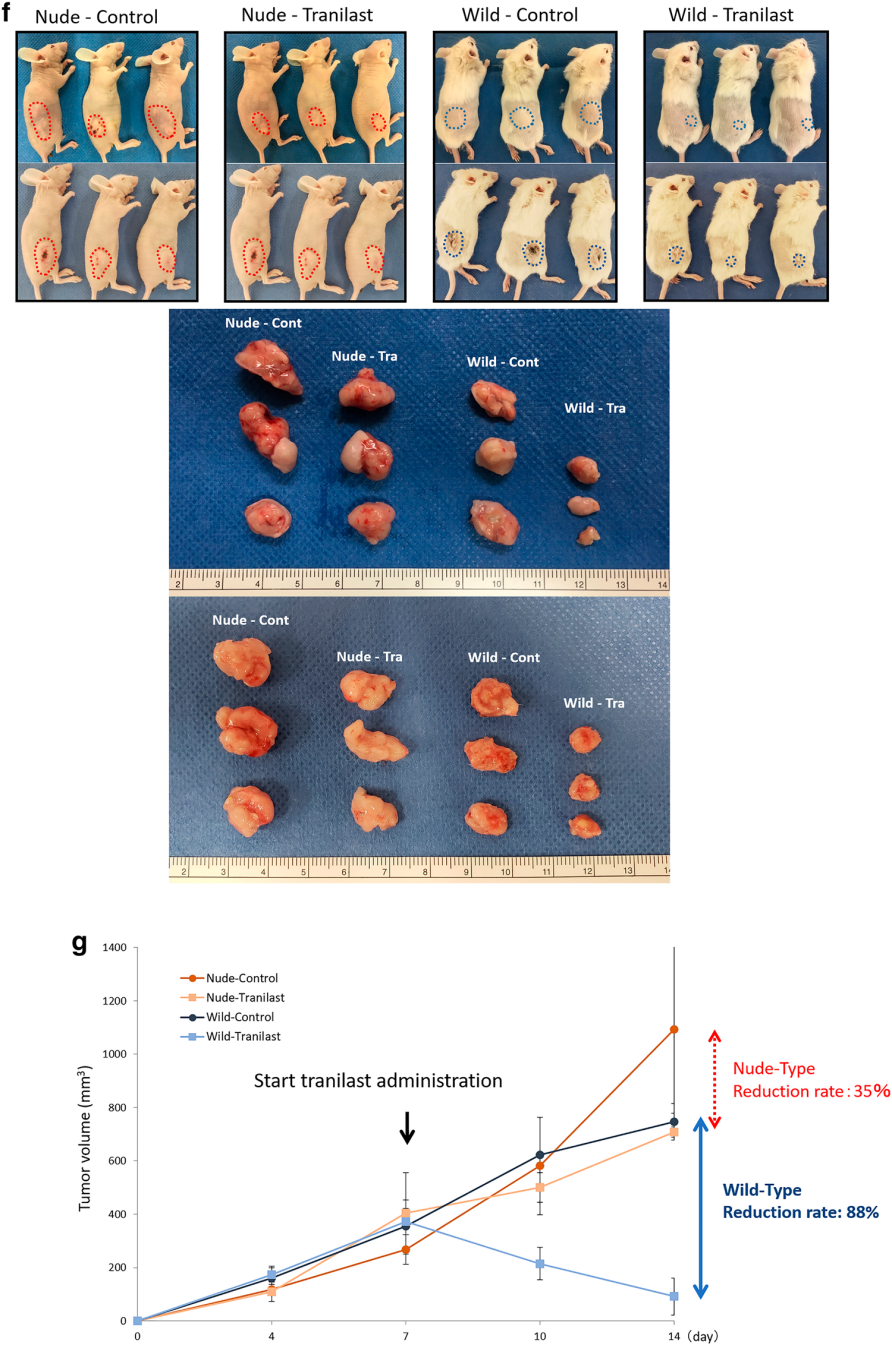
The anti-tumor effects of Tranilast administration using a mouse allograft model. **a** Representative macroscopic views of peritoneal nodules (arrows & circle) showing the two patterns in the control and Tranilast-treated allograft models on day 21. Results are expressed as the mean ± SEM (*n* = 6, **p < 0.01). Microscopic views of peritoneal tumors. Fibrotic tissues were determined by Azan staining (**b**) and immunohistochemical examination of *α*-SMA (**c**). Fibrotic area and *α*-SMA-positive area were measured and are shown as a percentage (fibrotic area, *α*-SMA-positive area, or whole section area). Data are expressed as the mean ± SEM of five representative regions at × 200 high-power magnification (*n* = 6, **p < 0.01). **d** Microscopic view of subcutaneous tumors. Fibrotic tissue was determined using Azan staining. Data are expressed as the mean ± SEM of five representative regions at × 200 high-power magnification (*n* = 6, **p < 0.01). **e** Vessel area was determined using CD31 immunostaining. Data are expressed as the mean ± SEM of five representative regions at × 100 high-power magnification (*n* = 6, **p < 0.01). **f** Macroscopic image of the subcutaneous tumor (six mice in each group). *Tra * Tranilast, *Cont* control. **g** The mean volume of the subcutaneous tumors co-inoculated with YTN16 and LmcMF cells was evaluated until 14 days after inoculation (*n* = 6). In wild-type BALB/c mice, the reduction rate in the Tranilast-treated group was 88%, while in nude BALB/c mice, the reduction rate in the Tranilast-treated group was only 35%

We evaluated the anti-tumor effects of Tranilast administration using a mouse allograft PM model. Tumor weight was significantly lower in Tranilast-treated peritoneal tumors than that in the untreated group (1.59 ± 0.2 g vs. 0.81 ± 0.2 g, *p* < 0.01) (Fig. 3a). In comparisons using Azan staining, the fibrosis area in Tranilast-treated tumors was smaller than that in the untreated group (3.7 ± 0.4% vs. 12.2 ± 1.3%, *p* < 0.01) (Fig. 3b). The area stained with *α*SMA, which indicates activated myofibroblasts, was 3.6% in tumors with Tranilast and 10.6% without Tranilast (*p* < 0.01) (Fig. 3c).

Next, we compared the anti-tumor effects of Tranilast in immunocompetent and immunodeficient mice using subcutaneous tumor models of BALB/c and BALB/c-nu/nu mice. The reduction in the fibrosis area in Tranilast-treated tumors in BALB/c and BALB/c-nu/nu mice was comparable (4.79 ± 0.80% vs. 5.01 ± 1.62%) (Fig. 3d). We then compared the effects of Tranilast on intratumoral angiogenesis by CD31 immunohistochemical staining. The vessel areas between the subcutaneous tumors in BALB/c and BALB/c-nu/nu mice were comparable (4.46 ± 0.61% vs. 4.85 ± 0.77%). Although Tranilast significantly inhibited angiogenesis in both mice (*p* < 0.01), the reduction effects of their vessel area were equivalent (0.75 ± 0.16% vs. 1.10 ± 0.57%) (Fig. 3e).

However, the reduction in tumor volume in BALB/c-nu/nu mice was 35% in the Tranilast-treated group compared to the untreated group, whereas in BALB/c mice, the reduction rate was 88% in the Tranilast-treated group (Fig. 3f, g), indicating a significant anti-tumor effect in immunocompetent mice.

### Analysis of immune microenvironment in mouse PM tumor and mouse subcutaneous tumor

**Fig. 4:**
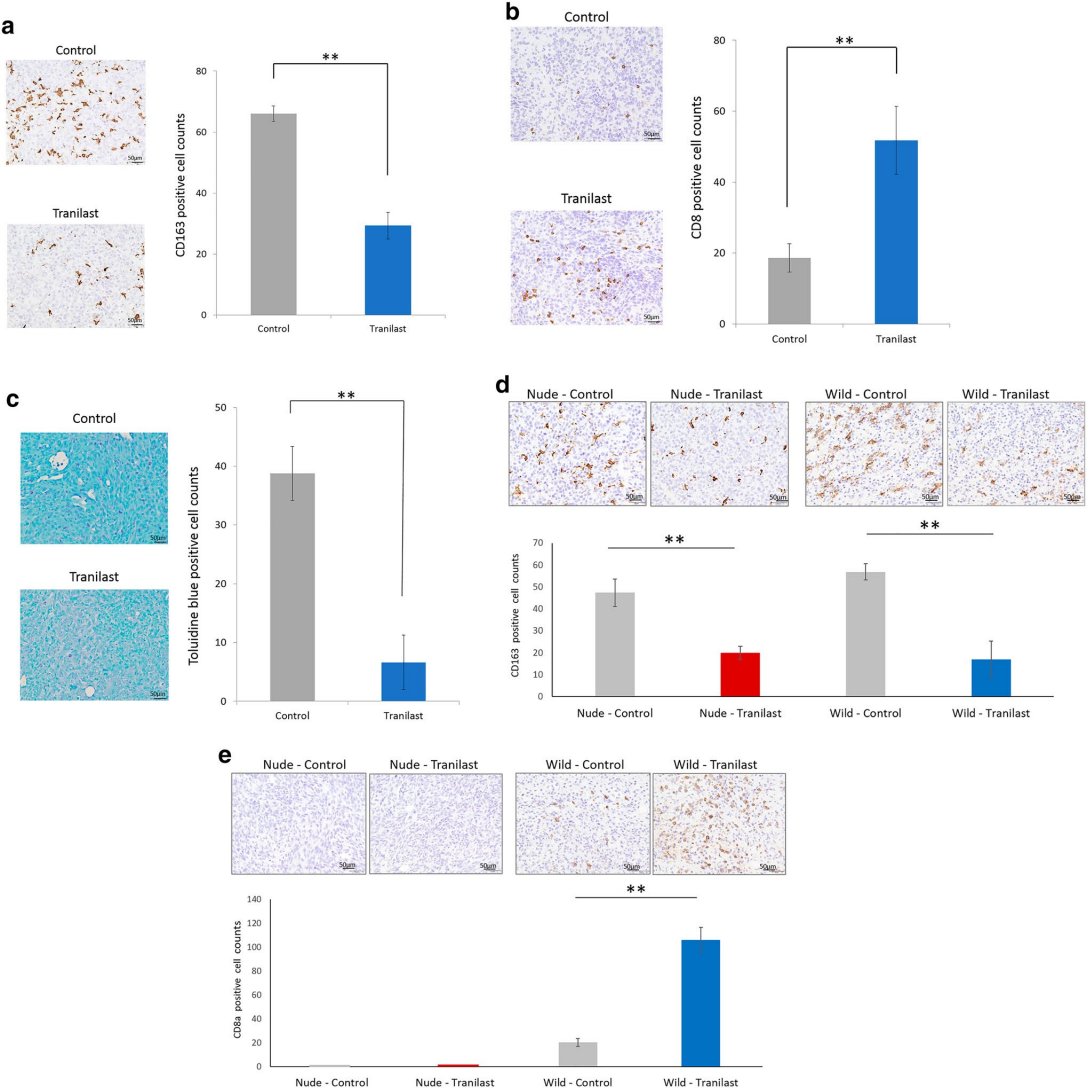
The effect of Tranilast on immunomicroenvironment in mouse peritoneal and subcutaneous tumors. Representative microscopic image of CD163^+^ cells (**a**), CD8^+^ cells (**b**), and mast cell (**c**) infiltration in each tumor of peritoneal allograft models (original magnification × 200). CD163^+^ and CD8^+^ cells were determined immunohistochemically, and mast cells were stained with toluidine blue. The number of infiltrated cells was measured and is shown as the average count in five non-overlapping tumor areas. Results are expressed as the mean ± SEM (*n* = 6, **p < 0.01). Representative microscopic images of CD163 + (**d**) and CD8 + cell (**e**) infiltration of each tumor in subcutaneous models (original magnification × 200). Results are expressed as the mean ± SEM (*n* = 6, *p < 0.05, **p < 0.01)

In the mouse PM tumors, there was no significant difference in the CD68^+^ cell (M1 macrophage) counts and CD4^+^ cell counts between tumors treated with and without Tranilast (data not shown). In contrast, fewer CD163^+^ cells (M2 macrophages) were found in Tranilast-treated tumors than untreated tumors (29.3 ± 4.4 cells vs. 66.0 ± 2.4 cells, *p* < 0.01) (Fig. 4a). Significantly higher infiltration of CD8^+^ cells was found in Tranilast treated-tumors than untreated tumors (50.5 ± 9.6 cells vs. 18.6 ± 3.9 cells, *p* < 0.01) (Fig. 4b). Mast cells were recognized by toluidine blue staining; significantly lower infiltration was found in Tranilast-treated tumors than untreated tumors (6.6 ± 4.6 cells vs. 38.8 ± 4.6 cells, *p* < 0.01) (Fig. 4c). In the mouse subcutaneous tumors, Tranilast-treated tumors showed decreased infiltration of CD163^+^ cells compared with untreated tumors in both BALB/c and BALB/c-nu/nu mice (Fig. 4d). In addition, CD8^+^ cells were found to be significantly higher in Tranilast-treated tumors than in untreated tumors (Fig. 4e).

### Analysis of apoptotic cells in mouse PM and subcutaneous tumors

**Fig. 5:**
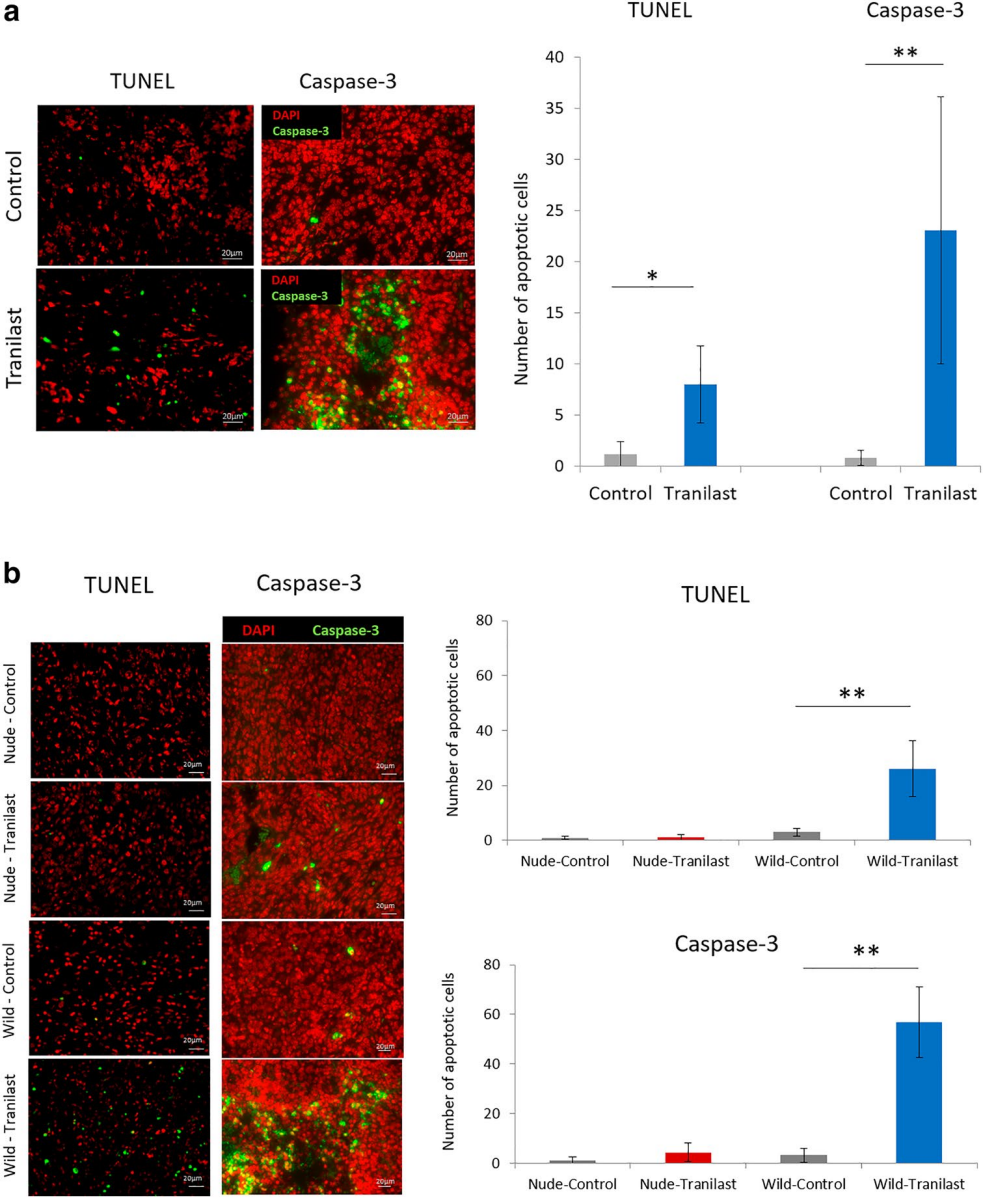
The effect of Tranilast on tumor cell apoptosis in mouse peritoneal and subcutaneous tumors. Representative image of immunohistochemical analysis of apoptosis using TUNEL and active caspase-3 immunofluorescence (original magnification × 100). The number of apoptotic cells in peritoneal tumors (**a**) and subcutaneous tumors (**b**) was measured and are shown as the average count of five non-overlapping tumor areas. Results are expressed as the mean ± SEM (*n* = 6 in each group for peritoneal tumors and *n* = 6 in each group for subcutaneous tumors, *p < 0.05, **p < 0.01)

We evaluated the apoptosis of tumor cells by TUNEL assay and active caspase-3 staining. In C57BL/6 J mouse PM tumors, the number of apoptotic cells was significantly higher in Tranilast-treated tumors than untreated tumors (TUNEL: 1.2 ± 1.2 cells vs. 8.6 ± 3.8 cells, *p *= 0.026; Active caspase-3 staining: 0.8 ± 0.7 cells vs. 23.1 ± 11.8 cells, *p *< 0.01) (Fig. 5a). Similarly, in BALB/c mouse subcutaneous tumors, the number of apoptotic cells was significantly increased in the Tranilast-treated group compared to the untreated group (TUNEL: 3.4 ± 2.3 cells vs. 27.5 ± 10.6 cells, *p *< 0.01; Active caspase-3 staining: 3.3 ± 2.8 cells vs. 54.8 ± 14.2 cells, *p *< 0.01). In contrast, in BALB/c-nu/nu mice, no increase in the number of apoptosis cells was observed in the Tranilast-treated group (TUNEL: 0.9 ± 0.8 cells vs. 1.2 ± 0.7 cells; Active caspase-3 staining: 1.4 ± 0.4 cells vs. 4.4 ± 3.7cells) (Fig. 5b).

## Discussion

Although CAFs have been implicated as key players in the immune suppressive microenvironment, the treatment strategies to improve their characteristic TMEs are largely unknown. Here, Tranilast was found to downregulate the CXCL12 expression in LmcMF as CAFs, resulting in decreased M2 macrophage migration in vitro. In addition, we showed decreased infiltration of M2 macrophages into peritoneal tumors co-inoculated with YTN16 and LmcMF in C57BL/6J mice. Interestingly, no downregulation of chemokines other than CXCL12 was found by Tranilast administration, though it is well known that CCL2 (MCP-1) is a major chemoattractant for recruiting monocytes, causing increased CCR2-expressing macrophage migration into the tumor site [22, 23].

Recently, it has been reported that the anti-alcoholism drug disulfiram acts as a potent inhibitor of macrophage accumulation in tumors via direct binding to a specific site of the chemokine receptor-binding domain of FROUNT [24]. Clinical trials using this drug are currently ongoing as a repositioned drug with anti-cancer effects [25]. However, this clinical study was performed based on the results of preclinical mouse models using subcutaneous tumors without stromal tissue, including CAFs, which differ from the real clinical TME. In contrast, our mouse model has rich stroma tissues, including CAFs, extracellular matrix, inflammatory cells, and vessels, similar to clinical features. Using a nude mouse model, it was impossible to analyze the interaction between lymphocytes and macrophages.

In our allograft PM model using immunocompetent mice, Tranilast increased the infiltration of CD8^+^ cells in addition to the inhibition of M2 macrophage migration. Furthermore, Yamaguchi et al. found that M2 macrophages secrete various cytokines, such as vascular endothelial growth factor (VEGF), IL-10, amphiregulin, and matrix metalloproteinase 1 (MMP-1) [26]. These cytokines exhaust CD8^+^ cells directly or indirectly, and enhance both the invasion and proliferation of cancer cells. Thus, inhibition of M2 macrophage migration into the tumor region could increase CD8^+^ cell infiltration and decrease tumor progression.

Tranilast has also reduced the accumulation of mast cells that express CXCR4 in peritoneal tumors by suppressing CXCL12 secretion from CAFs. Mast cells secrete various chemical mediators, including TGF-β and IL-17A. We have previously demonstrated that Tranilast decreases the degree of fibrosis in peritoneal tumors by inhibiting mast cell migration and suppressing mast cell degranulation [11]. Both TGF-β/Smad and IL-17A/STAT3 signals from mast cells induce CAF activation, resulting in the upregulation of cytokine/chemokine secretion, including CXCL12 and ECM production, including type I collagen. One possibility is that infiltration of CD8^+^ cells into the tumor may have been impeded by systemic circulation due to the high intratumoral pressure associated with tumor fibrosis and anticancer drugs. However, this hypothesis can be rejected because CD4^+^ cells were not inhibited in infiltration into fibrous tumors. Accordingly, Tranilast reduced migration of immune-suppressor cells by downregulating CXCL12 expression; thus, antitumor immunity was recovered through the induction of CD8^+^ cell infiltration. The immunogenic growth inhibition effect of CD8^+^ cells as cytotoxic T cells (CTLs) was confirmed because Tranilast-treated tumors showed many apoptotic cells. In our subcutaneous model comparing BALB/c and BALB/c-nu/nu mice, tumor volume was significantly reduced in BALB/c mice. Although fibrosis, angiogenesis, and CD163 + cells (M2 macrophages) infiltration were reduced in both tumors, the number of CD8 + cells and apoptotic cells were significantly increased only in Tranilast-treated BALB/c mice. These results indicate that the difference in the tumor reduction rate was affected by the cytotoxic effect of CD8^+^ cells in the BALB/c model.

In this study, we administered oral Tranilast at a dose 200 mg/kg/day of in vivo, which revealed no body weight loss compared to the control without Tranilast. The tissue concentration of Tranilast is approximately 75 mM after oral administration, which is only about twofold higher than the clinical dose [18]. As the body weight loss of peritoneal tumor-bearing mice was > 10% throughout the experiment in each group, 75 mM of Tranilast was considered non-toxic. Therefore, Tranilast could be a promising candidate for the treatment of PM in GC by drug repositioning.

This study had several limitations. First, only one cell line, YTN-16, was used as a mouse-derived gastric cancer; however, there were no other cell lines except for sublines producing hepatic metastasis or lymph node metastasis. Second, this PM model did not show PD-L1 expression in YTN16 (data not shown). Even if cancer cells express PD-L1, we believe that Tranilast with ICI treatment can achieve favorable results.

By suppressing the activity of CAFs, Tranilast inhibits the migration of M2 macrophages and mast cells, which produce various cytokines that deplete CTLs. By inhibiting the TGF-β/Smad signal, Tranilast also reduces Foxp3^+^ regulatory T cells [27] and suppresses EMT, which decreases invasion ability, eliminates drug resistance, and suppresses fibrosis, resulting in increased CD8^+^ cell infiltration. In conclusion, Tranilast has multimodal effects that play important roles in the inhibition of tumor progression by improving the tumor immune microenvironment. Thus, Tranilast could be a promising repositioning drug for gastric cancer treatment, especially for peritoneal metastasis.
